# Association of superoxide dismutase enzyme with staging and grade of differentiation colorectal cancer: A cross-sectional study

**DOI:** 10.1016/j.amsu.2020.08.032

**Published:** 2020-09-02

**Authors:** Warsinggih, Budi Irawan, Ibrahim Labeda, Ronald Erasio Lusikooy, Samuel Sampetoding, M. Ihwan Kusuma, Julianus Aboyaman Uwuratuw, Erwin Syarifuddin, Prihantono, Muhammad Faruk

**Affiliations:** a Division of Digestive, Department of Surgery, Faculty of Medicine, Hasanuddin University, Makassar, Indonesia; b Division of Oncology, Department of Surgery, Faculty of Medicine, Hasanuddin University, Makassar, Indonesia; c Department of Surgery, Faculty of Medicine, Hasanuddin University, Makassar, Indonesia

**Keywords:** Antioxidant, Neoplasm grading, Superoxide dismutase, Colorectal cancer

## Abstract

**Introduction:**

The increase of superoxide dismutase (SOD) level in colorectal cancer (CRC) patients based on the examination of staging and grade of differentiation still evidently represents a clinical problem. SOD level raises at a certain staging and reduce at a certain grade of differentiation. For that reason, this study aimed to assess the association between SOD and the variables analyzed in this study.

**Materials and methods:**

This study was observational study using a cross-sectional research design aimed to measure the association between SOD and staging as well as grade of differentiation in CRC incidence. The study was conducted in our institution from January until March 2018.

**Results:**

Statistical analyses of the data derived from the laboratory indicated that age and histopathological examination (TNM staging) had statistically significant correlation with SOD1 level. This significant correlation was proven from results of the statistical analyses of each variable at p = 0.039 (age) and p = 0.001 (TNM staging) respectively. Subsequent tests concerning the correlation between age and TNM staging on SOD1 level revealed that the study samples in the category of 30–49 age years old showed statistically significant correlation with SOD1 level with p = 0.009.

**Conclusion:**

The increase of grade of differentiation was proportional to the increase of SOD1 level as antioxidant against cancer in CRC patients.

## Highlights

• The increase of SOD level is proportional to the increase of severity level of colorectal carcinogenesis.
• SOD1 level can be used as a valuable biomarker to detect cancer in the human colon.
• Age and TNM staging had significant association with SOD1 level among the colorectal cancer (CRC) patients.

## 1 Introduction

Colorectal cancer (CRC) is a common malignant disease in Western countries [1]. Morbidity and mortality statistics of CRC persistently rise all over the world, including in Indonesia [2]. In the United States of America, CRC is the third deadliest cancer (regardless of gender) behind lung cancer and prostate cancer of the male group [3]. There are more than 130 thousand new cases of CRCs in the USA, with more than 49 thousand cases lead to deaths in 2016 [4]. According to data from the Globocan 2018, the overall incidence rate of CRC in Indonesia was 12.8 per 100,000 adults with 9.5% mortality of all cancer cases in this country [2,5]. Several known causal factors that lead to the ascending trend of CRC and mortality in the developed countries are higher socioeconomic status, dietary behaviors, inappropriate health activities and unhealthy lifestyles among certain populations [6].

Several population groups have higher risk of CRC that include populations with history of adenomatous polyps [7], family history with CRC [8], inflammatory bowel disease (IBD), genetic risks (FAP and lynch syndrome), high-fat diet (particularly high intake of animal foods) [6,9,10], inappropriate physical exercises [11], obesity [12], smoking habits [13] and heavy alcohol consumption [6,12]. Current studies are focusing the research of gene alterations or gene mutations responsible for the most forms of inherited CRC that affect the disorders of process in cells.

Oncogenetic molecular variables including DNA ploidy, expression of oncogene and anti-oncogene (mutation of Ki‐ras oncogene, mutation of Deleted in Colorectal Carcinoma/DCC, p53, nm23 gene and other allele aberrations), tumor-related antigen and other immunological factors (carcinoembryonic antigen/CEA, Mucin-associated antigen, Human leucocyte antigen DR/HLA-DR and The epidermal growth factor receptor/EGFR) are thought to play important roles at different stages of colorectal carcinogenesis [[14], [15], [16]]. It is known that a main feature of cancer cells is the action of reactive oxygen species (ROS) that responsible for carcinogenesis in which high levels of intracellular ROS and an abnormal regulation of ROS mediate adverse pathological conditions such as angiogenesis and cancer [17]. One of these reactive oxygen species is superoxide anion (O2−) that activates carcinogenetic and has a significant adverse effect that triggers mutations in tumor suppressor genes leading to DNA damage [18].

The human body defense system produces superoxide dismutase-1 (SOD1) that breaks superoxide anions to halt the process of carcinogenesis [17,19]. The increase of SOD level is proportional to the increase of severity level of colorectal carcinogenesis as shown in cancer tissues compared to normal tissues [20]. The increase of SOD enzyme in CRC patient groups than the control group is mainly found at both staging I and III, whereas, SOD level is lower at staging IV in CRC patients. SOD enzyme levels increase among all the CRC patients than the control group [21].

The inconsistency of previous studies concerning SOD levels in plasma in colorectal incidence prompts the authors of this study examine the association between SOD and several staging and grade of differentiation in CRC patients.

## 2 Materials and methods

### 2.1 Methods of the study

This study was observational study using a cross-sectional analysis design aimed to measure the association between SOD and several variables that lead to CRC incidence. This study was conducted at our institution from January until March 2018. Examinations of blood samples used the ELISA method at the accredited laboratory. All experimental procedures for the treatment of patients as the study samples were reviewed and approved by our research Ethics Committee no. 339/H4.8.4.5.31/PP36-KOMETIK/2018. This works has been reported in line with the STROCSS criteria [22]. This study is registered with the Research Registry and the unique identifying number is: 5730.

### 2.2 Population and samples

The population in this study were on all CRC patients that were treated in our institution. The time since the diagnosis of cancer varied from a minimum of two months to more than one year. Cancers were diagnosed by histopathological examination.

Collection of the samples was performed according to the consecutive sampling method that includes all the CRC patients who fulfilled the criteria as specified in the following inclusion and exclusion criteria. Inclusion criteria: Subject were diagnosed with CRC by histopathological examination, subject were not diagnosed with acute or chronic disease infections, subject did not have history of hemostatic disease, subject was under good nutritional status, and subject or subject legal representative had signed the informed consent form. Exclusion criteria: subject with CRC that underwent chemotherapy, radiotherapy or chemoradiotherapy, and subject with CRC that had malignant tumors in other organs.

The blood samples were collected (4–5 mL) through peripheral vein. The blood serum was separated in a plain vacuum tube, aliquoted and stored at −20 °C and used for the following assays of SOD.

### 2.3 Enzyme assay

Human Superoxide dismutase 1 — SOD1 activity was measured spectrophotometrically using Sandwich ELISA (enzyme-linked immunosorbent assay) kit the Human Super Oxidase Dismutase-1 (SOD1) (Cat. No LS-F5770-1, LifeSpan Biosciences, Inc. USA) in accordance with the kit protocol. Absorbance readings were obtained using an ELISA Thermo Scientific™ Multiskan™ FC Filter-based Microplate Photometer (Thermo Fisher Scientific, USA) at 450 nm wavelength.

### 2.4 Data analysis

The collected data were analyzed and processed using SPSS for Windows 22 software (IBM SPSS Statistics for Windows, Version 22.0. IBM Corp., Armonk, NY). The statistical test of the data to determine the association between SOD and the variables of CRC incidence used the Kruskal-Wallis test.

## 3 Results

Based on the data derived from the medical record at our institution, a total of the study samples were 34 CRC patients who fulfilled the inclusion and exclusion criteria as specified in this study. Table 1 indicated that the diagnosed CRC patients were in mean age 43.29 ± 11.851 years (range 14–61). In terms of gender, the CRC patients for the male group were 73.5% or 25 patients of the total 34 samples collected in this study. In terms of suffering period of CRC, there were 55.9% or 19 patients with CRC for the period of 6–12 months. Based on the histopathological examination (TNM staging), the staging IV of CRC was the greatest number of sufferers amounted to 14 patients or 41.2%, whereas, in terms of grade of differentiation, there were 21 patients or 61.8% were in poor grade during the study period.

**Table 1:** Baseline characteristics of the study samples.

Variable	Category	n	%
Gender	Male	25	73.5
	Female	9	26.5
Age (years)	<30	6	1.7.6
	30–49	16	47.1
	≥40	12	35.3
Suffering period (months)	<6	8	23.5
	6–12	19	55.9
	>12	9	20.6
Histopathological results (TNM)	Staging 1	0	0
	Staging 2	11	32.4
	Staging 3	9	26.5
	Staging 4	14	41.2
Differentiation	Good	3	8.8
	Moderate	10	29.4
	Poor	21	61.8

Table 2 showed results of the data analyses concerning the association between SOD1 and gender using the Mann-Whitney's test and showed that there was not statistically significant correlation between gender and SOD1 in CRC incidence with p = 0.740. The statistical analysis was conducted using the Kruskal-Wallis test to determine the correlations among the study variables. Results of the Kruskal-Wallis test revealed that age had statistically significant correlation with SOD1 in CRC incidence with p = 0.039.

**Table 2:** Associations between SOD1 and the specified variables of the study.

Variable	n	Mean ± SD	*p-*value
Gender			
Male	25	2158.0 ± 1075.6	0.740^a^
Female	9	2299.0 ± 1058.6	
Age (years)			
<30	6	1543.4 ± 787.0	0.039^b^
30-49	16	2713.5 ± 982.5	
≥40	12	1830.5 ± 992.9	
Suffering period (months)			
<6	8	2122.8 ± 1102.4	0.987^b^
6-12	19	2206.7 ± 1099.9	
>12	7	2247.5 ± 1042.1	
Histopathological results (TNM)			
Staging 2	11	1440.4 ± 694.8	0.001^b^
Staging 3	9	1854.9 ± 821.6	
Staging 4	14	3007.5 ± 880.9	
Differentiation			
Good	3	2542.9 ± 283.5	0.454^b^
Moderate	10	1816.9 ± 817.9	
Poor	21	2325.9 ± 1194.3	

a Mann-Whitney's test.

b Kruskal-Wallis test.

Suffering period of CRC did not show statistically significant correlation between SOD1 and CRC incidence with the statistical test value of p = 0.987, although the mean value ± SD in the age category of 6–12 months was 2206.7 ± 1099.9. Histopathological examination (TNM staging) showed statistically significant correlation with SOD1 in CRC incidence at p = 0.001. Therefore, histopathological examination had statistically significant effect to CRC incidence. Differentiation was the last variable tested to determine the association between SOD1 and CRC. Results of data analyses using the Kruskal-Wallis test showed that there was not statistically significant correlation between differentiation and SOD1 at p = 0.454. Since the critical limit value was p > 0.05, it was concluded that SOD1 did not have significant correlation with differentiation in CRC incidence among all the study samples at our hospital.

**Fig. 1:**
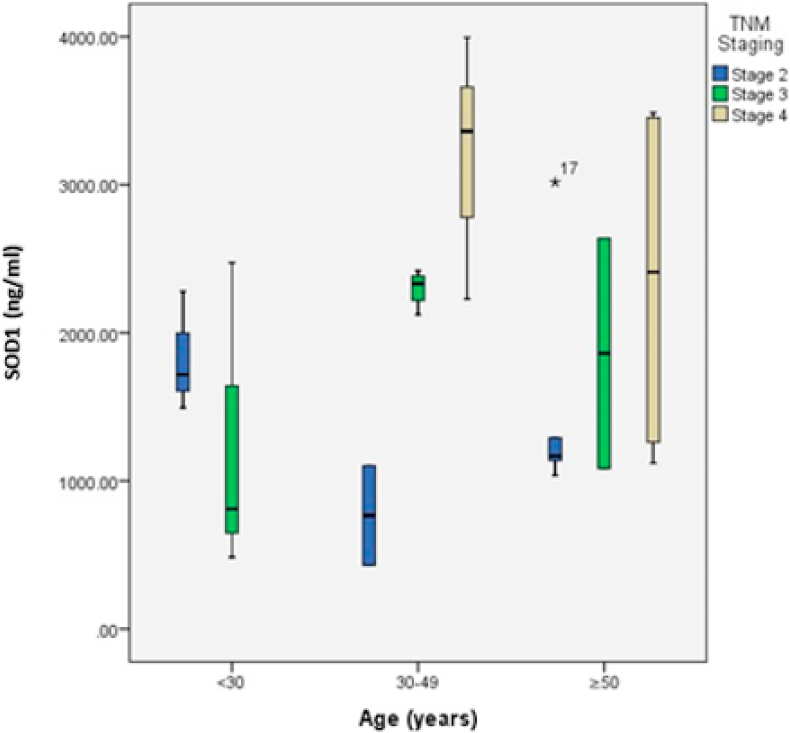
SOD1 serum levels based on TNM staging.

Due to two variables (age and TNM staging) were correlated with SOD1 in CRC incidence, statistical tests were conducted to determine the association between the two variables (age and TNM staging) and SOD1 in CRC incidence (Fig. 1). Table 3 presented results of the statistical data analyses concerning the correlation between TNM staging and age with SOD1 in which the greatest number of patients who susceptible to CRC incidence was in the category of 30–49 years old with the histopathological examination (TNM staging) at staging 3 that showed significant correlation. This significant correlation was proven by p = 0.009.

**Table 3:** Associations between SOD1, TNM staging and age.

Age (years)	TNM	n	Mean ± SD	*p*-value
<30	Staging 2	3	1830.7 ± 405.0	0.513^a^
	Staging 3	3	1256.1 ± 1066.2	
30–49	Staging 2	2	766.7 ± 474.0	0.009^b^
	Staging 3	4	2301.0 ± 127.0	
	Staging 4	10	3267.8 ± 565.0	
≥50	Staging 2	6	1469.8 ± 762.2	0.397^b^
	Staging 3	2	1860.8 ± 1098.3	
	Staging 4	4	2356.5 ± 1270.5	

a T-test.

b ANOVA test.

## 4 Discussion

The majority (82.4%) of CRC sufferers were aged 30 years and above, with characteristics similar to the report by Malinowska et al. [23]. This possibly results from the causal factor of aging, followed by the increasing exposure of individuals to free radicals. This leads to elevated oxidative stress, and a potential upsurge in cancer risk [24].

The majority of samples were male (73.5%), with a male-female ratio of 2.78: 1. This characteristic is congruent with the research conducted by Murphy et al. (2011) and Malinowska et al. [23], and is attributed to several environmental factors, including the relatively higher incidence of cigarette smoking and alcohol use in men. A recent study addressed that estrogen had protective effects against CRC [25].

Furthermore, most patients (67.7%) undergo treatments at an advanced stage (III-IV), which is in accordance with the “CRC Statistics” study conducted by Siegel et al., [4]. Based on this research, the outcome was due to the direct proportional relationship between low socio-economic levels and the availability of adequate health access [26]. Furthermore, the initial symptoms alongside other cancers are not specific, as some tend to display no symptoms, especially at early stages.

This study showed an increase in the SOD enzyme levels of CRC patients to an average of 2195.4 U/ml (normal value is 165–240 U/ml). Moreover, higher levels indicate more significant activity against oxidative stress responses in sufferers. This disease phenomenon is characterized by DNA damage, resulting in the genome instability for cancers known to attack humans. The body subsequently produces SOD enzymes as a backbone to primarily prevent oxidative stress from affecting cells [[27], [28], [29]].

In addition, there was also a statistically significant difference (p < 0.05) in relation to CRC stage, as higher amount of SOD enzyme was recorded in advanced levels, including stage III and IV. This shows a positive correlation between the disease progression and the oxidative stress magnitude. The findings are congruent with Malinowska et al., where the enzyme levels increased from the non-invasive to metastatic advanced stages [23]. Theoretically, an over-expression observed in the latter phase of the disease, is related to the ability to catalyze O2-toxic into H2O2, including reactive oxygen species (ROS), known to play a role in oxidative stress [24,[30], [31], [32]].

Based on histopathological images, the highest SOD enzyme levels were obtained from the WHO type I, and III, followed by II adenocarcinoma, characterized by a moderate degree of differentiation. This statistically proves the absence of no significant differences between the levels in CRC sufferers with WHO types I, II or III (p > 0.05). Furthermore, the results were observed to be different from Skrzycki [21], where healthy individuals demonstrated the highest SOD enzyme levels, followed by the images obtained from people with good, moderate, and the lowest adenocarcinoma, based on bad differentiation degree [33,34]. This explains the proportional relationship between lower enzyme activity and poor cell differentiation.

The formation of reactive oxygen species (ROS) is a normal consequence of various essential biochemical reactions in the body. Girgin et al., also proved the ROS formation on chronic diseases in the gastrointestinal tract, with phagocytes present in intestines as the main oxygen source [35]. These products tend to accumulate in the mucus of patients with intestinal disease, and also increase the incidence of cancer. Therefore, various anti-oxidant defense mechanisms inside and outside the cells are needed in the protection of cell components against oxidative damage, including the phospholipid membrane [[36], [37], [38]].

Numerous current studies aim to analyze the relationship and balance of oxidants and antioxidants towards the CRC stage. Another study reported on the simultaneous increase in SOD enzyme levels alongside the stage and differentiation of sufferers [[39], [40], [41]]. This is congruent with another literature said where higher degree of malignancy aligns with the SOD levels, and also with the depth and veins invasion [42]. Colorectal cancer is associated with DNA damage due to oxidative stress as well as increased oxidative damage to proteins and lipids [27,43].

In addition, an upsurge in the SOD levels is followed by elevated polyp diameter/magnitude, as well as the villous component and the polyp dysplasia degree in patients with adenoma polyps. This current investigation proved a simultaneous increase in with the advancement in stages, which was not congruent with the differentiation degree [44,45]. This elevation in SOD also causes a rise in the product yield, e.g., hydrogen peroxide, where some cancers in humans were estimated to produce large quantities of hydrogen peroxide [46].

The upsurge in SOD observed in both the tumor tissue and serum of many cancer patients, including sufferers of malignant melanoma [47], some brain tumor, myoma [48], colorectal [42] and lung cancers [49,50], and malignant pleural mesotheliomas [51,52]. The exact mechanism in certain human cases has not been known, and is assumed to result from activations in the intestinal epithelial cells by cytokines, including TNF and IL-1. This phenomenon is also attributed to the direct effect of increasing reactive oxygen species (ROS), where the body attempts to achieve balance in oxidant and antioxidant levels.

Due to the institution of this study at a referral hospital in eastern Indonesia, the patients observed were mostly at an advanced stage. Hence, the sample based on WHO Type I histopathologic results was not available.

## 5 Conclusion

SOD1 level can be used as a valuable biomarker to detect cancer in the human colon. The increase of CRC grade is proportional to the increase of SOD1 production as an antioxidant against cancer in the human body. Results of this study proved that the variables of age and results of the histopathological examination (TNM staging) had significant association with SOD1 level among the diagnosed CRC patients.

## Provenance and peer review

Not commissioned, externally peer reviewed.

## Ethical approval

All procedure for human experiment has been approved by Ethics Commission Faculty of Medicine, Hasanuddin University Number: 339/H4.8.4.5.31/PP36-KOMETIK/2018.

## Sources of funding for your research

No funding or sponsorship.

## Author contribution

WS, BI, IL, RL, Pri, SM, IK, Boy, and ER, wrote the manuscript and participated in the study design. WS, BI, IL, RL, Pri, and MF drafted and revised the manuscript. WS, BI, IL, RL, SM, IK, Boy, and ER performed treatment and surgery. WS, BI, IL, RL, Pri, and MF performed bioinformatics analyses and revised the manuscript. All authors read and approved the final manuscript.

## Registration of research studies

This study is registered with the Research Registry and the unique identifying number is: researchregistry5730.

https://www.researchregistry.com/browse-the-registry#home/?view_2_search=researchregistry5730&view_2_page=1.

## Guarantor

Warsinggih.

## Declaration of competing interest

The authors declare that they have no conflict of interests.
